# Towards ageing well: Use it or lose it: Exercise, epigenetics and cognition

**DOI:** 10.1007/s10522-017-9719-3

**Published:** 2017-06-17

**Authors:** Irene Maeve Rea

**Affiliations:** 1 School of Medicine, Dentistry and Biomedical Science, Queens University Belfast, Belfast, BT7 INN Northern Ireland, UK; 2 Northern Ireland Centre for Stratified Medicine, C-TRIC, Biomedical Research Sciences Institute, Ulster University, Altnagelvin Hospital, Londonderry, BT47 6SB Northern Ireland, UK; 3 Belfast Health and Social Care Trust, Belfast, Northern Ireland, UK

**Keywords:** Healthy ageing, Lifestyle, Exercise, Exercise-induced stress response, Epigenetics, Mitochondrial biogenesis, Cognition, Brain-derived neurotrophic factor

## Abstract

More and more people are living into the 90s or becoming centenarians. But, the gift of increased ‘age span’ seldom equates with an improved ‘health-span’. Governments across the world are expressing concern about the epidemic of chronic disease, and have responded by initiating policies that make prevention, reduction and treatment of chronic disease, a public health priority. But understanding, how to age long and well, with the avoidance of chronic disease and later life complex disease morbidity is challenging. While inherited genes have an undoubted role to play in the chance of maintaining good health or conversely a predilection to developing disease and chronic ill health, there is increasing evidence that behavioural and environmental life-style choices may contribute up to 50% of the variability of human lifespan. Physical exercise is readily available to everyone, and is a simple cheap and effective form of life-style intervention. Exercise appears to help maintain good health and to reduce the risk of developing chronic disease and ill health. Evidence suggests that physical activity improves well-being across many health domains through out life, continues to offer important health benefits in older age groups and tracks with a ‘healthy ageing’ profile. Although many of the molecular pathways remain to be fully identified, here we discuss how physical activity and exercise is understood to produce changes in the human epigenome, which have the potential to enhance cognitive and psychological health, improve muscular fitness, and lead to better ageing with improved quality of life in older age.

## Introduction

A sedentary life style is associated with a high incidence of chronic disease such as cardiovascular disease, metabolic syndrome, type 2 diabetes, cognitive decline, and cancer (O’Donaghue et al. 2016; Honda et al. 2016; Ekelund et al. 2015; Kohl et al. 2012; Lee et al. 2012). Physical exercise improves health by contributing to disease prevention and helping recovery from illness. It helps to make us fitter, influences our cognition and psychological health, and reduces our risk of developing illnesses such as diabetes and heart disease (Barnes et al. 2011; Wendell et al. 2014; Young et al. 2016). Exercise is a key factor in maintaining our functional autonomy and can protect us from sarcopenic loss of muscle mass and strength which occurs with increasing age, and which is a major contributor to the frailty syndrome (Walston 2012; Cruz-Jentoft et al. 2010; Morley et al. 2001).

Scientists have known that certain genes become active or quieter as a result of regular exercise, but just how a bike ride, a brisk walk or a run might translate into a healthy life has remained unclear, until relatively recently. It is now believed that the epigenetic tags added to DNA, such as methylation, offer an explanation of how exercise regimes, with or without dietary intervention, can cause modification in the genome-wide methylation pattern of DNA (Ziller et al. 2013; Lindahl 1981). These tags act as on/off switches for the genes and the addition or removal these tags by methylation/demethylation, acetylation/deacetylation or phosphorylation/dephosphorylation, (Cedar and Bergman 2009), allow gene expression and activity to be fine-tuned and rapidly modified in response to environmental triggers such as physical exercise, nutritional availability, psychological stress and other stressors (Sakuma and Yamaguchi 2012). Exercise can have a variable response on different groups of genes, with some genes being hypomethylated and others hypermethylated (Horsburgh et al. 2015). Depending on which genes are involved, these may affect our health and our risk of disease.

A number of studies have found that a single bout of exercise leads to immediate changes in the methylation pattern of certain genes in our DNA and affects the proteins that these genes express (Hamer et al. 2013). Exercise-related methylation change appears to be stronger among older people, with age accounting for 30% of the methylation variation according to Brown (2015). In a study of 90-year-old male physicians in USA, Yates et al. (2008) has shown that maintaining active exercise was an important contributor to good quality ageing for physicians who reached 90 years and beyond, in good health. Similar findings have been reported by Rea et al. (2016) who identified, that maintaining exercise and physical activity were important life-style behaviours, self-reported by the nonagenarian sibling participants of the **GE**netics of **H**ealthy **A**geing (GeHA) project (Franceschi et al. 2007a) and **B**elfast **E**lderly **L**ongitudinal **F**ree-living **A**geing **ST**udy (BELFAST) (Rea et al. 2015), as important contributors to their long and good quality ageing (Rea and Rea 2011, 2013). In their review of fifteen longitudinal studies with at least 5-year follow-up times and a total of 288,724 subjects, aged between 18 and 85 years, Reiner et al. (2013) showed that physical activity appeared to have a positive long-term influence on many diseases, including weight gain, obesity, coronary heart disease, type 2 diabetes mellitus, Alzheimer’s disease and dementia.

Regular physical exercise protects against the development of chronic disease and ill health and provides health benefits across many domains, improves quality of life (O’Donovan et al. 2017; Reiner et al. 2013; Lollgen et al. 2009; Wen et al. 2011; Knoops et al. 2004) and reduces mortality (Myers et al. 2015; Kokkinos et al. 2010). Here we summarise some of the evidence suggesting that engaging in exercise does influence genes involved in metabolism and muscle growth, cognition and psychological well-being dependent on the intensity and duration of the exercise, and that changes can and do contribute to an improved healthy ageing profile.

## Don’t just sit there, do it!

### Exercise initiates a cellular stress response

Regular exercise improves health and decreases the incidence of oxidative-stress-related disease. Paradoxically however, exercise also acts as a producer of increased antioxidants and acts as a stimulating stressor. This effect is thought to be the result of exercise-induced adaption, a type of hormesis (He et al. 2016).

In both aerobic and anaerobic exercise, mitochondria are re-energised. This results in increased reactive oxygen species (ROS), which can lead to oxidative stress-related damage to membrane lipids, DNA, nuclear cellular organelles and impaired muscle contractility (Zuo et al. 2015). Evidence indicates that the exercise-induced ROS and nitric oxide (NO) signaling pathways are important in the initiation of the molecular adaptions in skeletal muscle. The redox- sensitive molecular pathways include nuclear factor erythroid 2-related factor (Nrf2), a redox-sensing transcription factor that is a regulator of antioxidants. Nrf2 mediates the adaptive responses to exercise training (Gounder et al. 2012) and promotes the trans-activation of antioxidant genes, leading to improved muscle protection (Muthusamy et al. 2012). Another exercise adaption involves up-regulation of mitochondrial biogenesis via peroxisome proliferator-activated receptor-γ coactivator-1α (PGC-1α) gene expression (Steinbacher and Eckl 2015), which also negatively feeds back to control mitochondrial biogenesis through Nrf2. Earlier upstream signals such as mitogen-activated protein kinase (MAPK) and nuclear factor (NF)-κB, also regulate PGC-1α expression in a redox-sensitive mechanism.

Acute and chronic exercise up-regulates endogenous anti-oxidant capacity and activities in skeletal muscle, therefore enabling an improved capacity to decrease the adverse effects of increased ROS production. Therefore, low levels of ROS-induced adaptation process, evoked by exercise, create a system that is a type of hormesis (Ji et al. 2016).

### Remodeling of muscle through epigenetic modification

The maintenance and remodeling of muscle mass thorough adult life, is regulated by an interacting balance between the anabolic and catabolic activities of genes, within the muscle, and by energy production from the mitochondria. Epigenetic modification plays a major part in muscle metabolism, in the remodeling process and in the epigenetic change that allows gene expression and activity to be rapidly modified in response to daily environmental triggers such as physical activity, nutritional availability, oxidative stress and hormonal changes (Sakuma and Yamaguchi 2012).

### Muscle growth and the insulin-like growth factor 1 (IGF-1) pathway

Muscle growth is controlled by the IGF-1 signalling pathway which stimulates protein synthesis and muscle regeneration (Philippou et al. 2007; Musaro et al. 2001). The insulin-like growth factor 1 (IGF-1)-Akt pathway controls muscle growth via mTOR (mammalian target of rapamycin) and FOXO genes, with fine control of the pathways managed through epigenetic modifications by phosphorylation or acetylation. IGF-1 upregulation stimulates both proliferation of satellite cells and binding of IGF-1 to its membrane receptor. IGF-1-receptor binding leads to Akt pathway activation and a chain of phosphorylation and acetylation by acetyltransferase p300 and P300/CBP-associated factor (PCAF), that repress the transcription factors of the FOXO family, so allowing protein synthesis and transcription of muscle-specific genes in response to environmental triggers (Zhang et al. 2005; Stitt et al. 2004). Muscle cell terminal differentiation is regulated by transcription factors, myocyte-specific enhancer-binding factors (MEF-1 and MEF-2) (Buckingham 1994), again with histone-acetyltransferase transcriptional co-activators, CREB-binding protein (CBP)/p300, controlling the balance between cell proliferation and differentiation.

### Muscle remodeling and FOXO gene pathway

The FOXO gene family, are major players and regulators of muscle breakdown and act through the two major pathways of muscle degradation- the ubiquitin-proteosome pathway and the lysosomal autophagy pathway (Attaix et al. 2005; Gomes et al. 2001). Both of these pathways depend on epigenetic modification, which primes muscles for degradation, usually through acetylation by histone de-acetylases (HADS) (Hasselgren et al. 2007; Alamdari et al. 2013). Hence muscle remodeling and degradation triggered in response to exercise, inactivity or environmental factors are constantly in a state of dynamic change, which is edited and fine-controlled by processes involving epigenetic change.

### Exercise upregulates mitochondrial biogenesis

Muscles cannot work without efficient energy production, and energy depends on the integrity of mitochondria. With increasing age, the mitochondria become sluggish and this compromises energy production and contributes to poor muscle strength and sarcopenia. Part of this decline can be due to de-conditioning of the muscles due to reduced physical exercise, sedentary inactivity or reduced exercise capability secondary to illness, or nutritional compromise. So a vicious cycle develops with reduced physical activity producing muscles that become weaker, are infiltrated with fat cells, and show less efficient mitochondria energy production. Inactivity begets fat infiltration, obesity, insulin resistance, the metabolic syndrome and type 2 diabetes, all of which are associated with inflammation or ‘inflamm-ageing,’ (Franceschi et al. 2007b), which itself also contributes to mitochondrial dysfunction (Marcus et al. 2010).

### Exercise in human studies

Ling et al. (2007) and Ronn et al. (2008), studied methylation in the mitochondrial genes-NDUFB6 and COX71A, which influence the mitochondrial proteins in the electron energy chain. Both genes showed altered gene patterns in human skeletal muscle with the COX71A promoter showing increased methylation in muscle from elderly people, compared to young twins (Ling and Groop 2009; Ronn et al. 2008, 2013). Gluckman et al. (2009) followed with suggestive evidence that epigenetic mechanisms underpinned the metabolic syndrome and its association with increased cardiovascular disease risk.

Importantly, exercise appears to revitalise mitochondrial function in muscles in both young and older individuals. It not only improves muscle function but also quality of life, with exercise improving mitochondrial function in older individuals as much as in younger exercising individuals (Carter et al. 2015; Kang and Ji 2013; Joseph et al. 2012). In their study of healthy-exercising men, compared to healthy-but-inactive men, Barres et al. (2012) found that genes involved in energy metabolism-PGC-1α, PPAR-γ and PDK4-showed a changed methylation pattern, dependent on the exercise intensity. Muscle biopsies taken from men who cycled the hardest, showed the greatest change in demethylation in their genes. In a series of follow-on experiments, Lindholm et al. (2014), compared an exercised leg to the matching non-exercised leg in the same subjects. They identified three main clusters of gene networks including (1) those involved in transcription pathways, (2) structural genes involved in increasing muscle mass, and (3) those involved in metabolic gene pathways. Edgett et al. (2013) also showed that exercise intensity produced positive epigenetic changes in terms of mitochondrial biogenesis. Here healthy male subjects cycling at 133% peak power showed a post –exercise change in gene expression of PGC-1α and its regulators in skeletal muscle biopsies. Cycling increased PGC-1α more at 100% capacity compared to 73%, although super maximal exercise seemed to blunt the response. Brown et al. (2015) too noted that it was possible through a training programme, to induce exercise-related epigenetic methylation change at 5 exercise-associated imprinted loci, that DNA methylation decreased with exercise at 60% of loci, the change was accentuated in older people and interpreted these findings as meaning, that it may be possible to re-wind the so-called ‘epigenetic clock’ (Horvarth 2013) of cellular ageing. In a pooled study of middle-aged 60,000 white males (average age 59 years) with data collected between 1994 and 2012, O’Donovan (2017) concluded that an exercise programme of one or two sessions per week of moderate or vigorous-intensity physical activity may be sufficient to reduce risks for all-cause, cardiovascular and cancer. Rea et al. (2016), found that maintaining physical exercise and physical activities thorough out life was a major theme, self-reported by GeHA and BELFAST nonagenarians sibling pairs, as an important factor in their long and good quality ageing.

Sustained level of physical activity in older age has been associated with overall improved health in several studies (Knoops et al. 2004; Yates et al. 2008). The Cambridge University Study found that modest physical exercise prolonged life (Ekelund et al. 2015), with similar findings in another large pooled cohort analysis (Moore et al. 2012). In the English Longitudinal Study, Rowlands et al. (2014), showed that participants surviving over 8-year follow-up, showed improved healthy ageing including absence of disease, freedom from disability, high cognitive and physical functioning and good mental health. This group also emphasized, that there were significant health benefits for those who took up exercise relatively late in life, a finding also noted Hamer et al. (2013). Research has shown increased methylation change in >18,000 genes in an >65 year-old-age group of participants exercising in a 6 week programme. In a critical review of 25 studies of physical activity and its effect on of DNA methylation, Voisin et al. (2015), in their study, concluded that both acute and long-term exercise schedules changed methylation in a highly tissue and gene-specific manner. Almeida et al. (2014) too found on follow-up of a population-based 11-year-longitudinal study that sustained physical activity was associated with improved survival and healthy ageing in older men. The authors concluded that vigorous physical activity of as little as 150 min weekly, seemed to promote healthy ageing and should be encouraged when safe and feasible.

Robinson et al. (2017), in attempting to further define the molecular and physiologic results assessed the adaption effects of three different types of skeletal-muscle-exercise in young and older adults. They found that high impact interval training (HIIT), increased VO_2_ peak, insulin sensitivity, mitochondrial respiration, fat-free mass (FFM), and muscle strength in both young and older participants. In contrast, resistance training increased insulin sensitivity, and FFM but not VO_2_ peak or mitochondrial respiration. These authors concluded that that while strength training was effective at building muscle mass, high-intensity interval training (HIIT) yielded the biggest benefits at the cellular level. The younger volunteers in the interval training group saw a 49% increase in mitochondrial capacity, and the older volunteers saw an even more dramatic 69% increase. Interval training also improved volunteers’ insulin sensitivity, which indicated a lower likelihood of developing diabetes. These researchers therefore concluded that supervised HIIT appeared to be an effective recommendation to improve cardio-metabolic health parameters in older adults. HIIT produced a pattern of gene expression independent of age, a robust increase in transcriptional and translational regulation of muscle growth, induced a strong up regulation of mitochondrial proteins and improved the age-related decline in muscle mitochondria.

Exercise keeps you healthy; this oft-repeated phrase rings true. Muscles have a plasticity that adapts depending on whether we engage in exercise or remain sedentary, and that also depends on our nutrition and health issues. The health benefits of regular exercise and physical activity cross all the domains of health and illness. We ignore the evidence at our peril (Arem et al. 2015; Carter et al. 2015). We should listen to, and follow the advice—Don’t sit, just do it and it’s never too late to start (Hamer et al. 2013).

## More exercise more brain

Physical exercise is associated with positive neural functioning. Over the last 20 years, research in humans and rodents has shown beneficial effects of exercise on the brain including enhanced learning and memory, structural plasticity, and protection against neurodegenerative disorders (reviewed by Cotman and Berchtold 2002; van Praage 2009; Thomas et al. 2015).

In animal studies, the mechanisms, which seem to underlie improved cognition as a result of exercise, include neurogenesis, synaptogenesis and synaptic plasticity, (Garcia et al. 2012; Ferreiria et al. 2010; Lou et al. 2008; Farmer et al. 2004). Brain derived neurotrophic factor (BDNF), a molecule implicated in learning and memory, has been shown to be consistently up-regulated in the hippocampus, dentate gyrus and perirhinial cortex in response to treadmill running in animals (Gomes da Silva et al. 2012; Hopkins et al. 2011; Griffin et al. 2009; Vaynman et al. 2006) and associated with improved spatial learning and memory (Aguiar et al. 2011; van Praag et al. 2005). Blocking BDNF signaling by infusion of BDNF receptor antibody could reduce the effects of exercise-enhanced cognitive benefits according to Korol et al. (2013). Impaired control of neuronal activity, which exercise-induced BDNF expression mediates, has been associated with various neurological and psychiatric disorders.

Exercise stimulates histone acetylation. Multiple experiments in animal models confirm that the hippocampus and cerebellum, both areas involved in motor control and learning, show increased global acetylation of histone 3 after exercise. This can lead to selective transcription of specific genes such as BDNF (Tsankaova et al. 2006). Following on from these findings, Gomez-Pinilla et al. (2011) reported that acetylation occurred in promoter of the BDNF IV sequence and was highly increased by exercise. This group also noted that exercise increased the relative proportion of hippocampal AceH3 but did not affect histone H4 levels, suggesting that there was relatively specific action of exercise on histone H3, which facilitates BDNF transcription.

### Energy metabolism and BDNF epigenetics

According to Wallace and Fan (2010), the main proteins, which produce bioenergetics in the cell, may also directly modify the epigenome, with changes in energy metabolism instigating the epigenetic events. The results of proteomic studies have shown that most of the proteins up regulated by exercise are associated with energy metabolism (Ding et al. 2006), with the functions of these proteins on cognition and synaptic plasticity being achieved through BDNF up-regulation.

### Exercise and cognition in human studies

Following on studies showing that physical activity increases the expression of BDNF in the rat brain, a series of studies were undertaken in order to establish any link between BDNF and post-exercise enhancement of mood and cognitive functions in humans (Colcombe et al. 2003, 2006; Angevaren et al. 2008; Voss et al. 2013). In another human study, Zoladz and Pilc (2010) showed the effect of a single bout of exercise and training on the brain derived neurotrophic factor (BDNF) expression in the brain, muscles and in the blood, with improved functioning of the body, but suggested that further evidence was required to support this finding. In a range of studies to assess the importance of exercise in maintaining and supporting cognition, some research suggests that resistance training can promote cognitive and functional brain plasticity in seniors who appear to have developed mild cognitive impairment (Nagamatsu et al. 2012; Heyn et al. 2004). Similarly, Coelho et al. (2014) reported that acute aerobic exercise increased BDNF in elderly participants with Alzheimers disease. Etgen et al. (2010) reported a follow-up study of elderly participants enrolled between the years 2001–2003 and found a reduced incidence of cognitive impairment in those engaged in moderate to high intensity physical exercise. The effects of a short-term four week combination exercise training regime was investigated by Nouchi et al. (2014), who showed that combination exercise training improved executive functions, episodic memory, and processing speed compared to those attributes measured in participants still on the waiting list control group. This report was the first of a study to demonstrate the beneficial effects of short-term combination exercise training on diverse cognitive functions of elderly people.

Individual studies have shown a range of variable outcomes with respect to the type and role of exercise in maintaining or improving cognition. In attempting to assess any consistent effect, (Smith et al. 2010) carried out a meta-analysis of adult human studies conducted over the previous 45 years. He found a positive impact of exercise on neurocognitive performance that included improved attention and processing speed, executive function and memory. In their study of community-dwelling older adults, who were evaluated on tasks of executive functioning before and after a month-long strengthening, nonaerobic exercise program, Anderson-Hanley et al. (2010), reported that participants who engaged in such exercise showed significantly improved scores on Digits Backward and Stroop C tasks when compared to participants who remained on the exercise waiting list. In a later systematic reviews of physical activity and healthy ageing, including assessment of cognitive function, there were clear outcome findings suggesting that late-life physical activity is beneficial for older people, with a suggestion of a dose–response relationship, between physical activity and cognition (Carvalho et al. 2014). In a critical review of 25 studies of physical activity and its effect on of DNA methylation, Voisin et al. (2015) concluded that both acute and long-term exercise schedules changed methylation in a highly tissue and gene-specific manner. Gallaway et al. (2017) in their review, provided evidence of physical activity’s role in reducing the risks of Alzheimers disease, vascular dementia and mild cognitive impairment.

There is a very large amount of evidence to suggest that lack of exercise due to our increased sedentary behavior may be a risk factor for the development of age-related cognitive impairment. A review by Barnes and Yaffee (2011) summarised evidence which suggested that approximately 13% of Alzheimers disease world-wide may be attributable to sedentary behaviour, and suggested that a 25% reduction in sedentary behaviour could prevent up to 1 million cases of Alzheimers disease. Sedentary behaviour not only contributes to cardiovascular disease (Wendell et al. 2014), is a risk factor for both Alzheimers and vascular dementias, but also seems to directly affect neurological processes, by reducing neurogenesis, synaptic plasticity, neurotrophin production, angiogenesis and by increasing inflammation (reviewed by Voss et al. 2014).

Exercise is a non-pharmacological life-style factor, which plays an important role in maintaining a healthy brain thorough out life and in human ageing. It is a powerful environmental intervention capable of gene expression change, improved neurogenesis, enhanced synaptic plasticity and signaling pathways, and involving epigenetic regulation in the brain and cerebellum in humans (Raji et al. 2016; Pareja-Galeano et al. 2014; Ling and Ronn 2014; Thomas et al. 2012; Erickson et al. 2011; Kempermann et al. 2010). These studies add to the accumulating evidence that exercise if good for everyone (Ploughman 2008), irrespective of age and may also keep our brains in sharper function by stimulating and maintaining neurogenesis and our cognition.

### Well-being

Being active is great for physical health and fitness and evidence shows that it can improve mental wellbeing as well. While most people can recognise mental wellbeing, as feeling good about oneself and about the world around one, there have been rather fewer attempts at how to define well being and even fewer attempts to measure it (Dodge et al. 2012). The 2010, World Health Organisation (WHO) definition states that ‘Health is a state of complete physical, mental and social well-being and not merely the absence of disease or infirmity.’

In an early review, and one of the few which has attempted to measure the association between well-being and exercise, Scully et al. (1998) critically examined the contention that the psychological benefits of exercise may equal, if not out-way, the physiological benefits, and gave guarded support for the role that exercise can play in the promotion of positive mental health. Later, Penedo and Dahn (2005) in their review evaluating the relationship between exercise, physical activity and physical and mental health in cross-sectional and longitudinal studies, as well as randomized clinical trials, showed that a growing literature found evidence to suggest that participants involved in exercise, physical activity and physical-activity interventions showed better health outcomes, including better general and health-related quality of life, better functional capacity and better mood states. In the 2009, Cochrane Review, Mead et al. (2009), concluded that regular moderate exercise may boost wellbeing, although the authors noted that the methodological quality of studies was variable.

The powerful influence of exercise on biological adaption seems likely to improve stress management. Rodrigues et al. (2015), reported that exercised rats showed no changes in DNA methylation when placed in a stress environment, whereas stress treatment produced a decrease in global DNA methylation in the hippocampus, cortex and periaqueductal grey matter in sedentary non-exercising animals. These authors considered that physical exercise had the potential to modulate changes in DNA methylation and gene expression secondary to stress treatment, with the epigenetic effect counteracting a negative experience. A large body of evidence in human participants suggests that exercise contributes to promoting mental health and wellbeing and contributes to alleviating the effects of depression (Gartlehner et al. 2015; Kandola et al. 2016), anxiety, schizophrenia, cognitive impairment and drug addition (for review see Duman et al. 2007). Although DNA methylation patterns at specific genetic loci may remain stable over long periods, they may fingerprint a predilection to disease development at a later stage. There therefore is the interesting possibility that reversal of disease or delay progression may be possible, since DNA methylation is a dynamic and reversible process (review Karpova 2014). Although current research opens novel directions for the discovery of new promising future therapeutic targets for treatment of psychiatric disorders, the present-day evidence firmly supports a role for exercise and physical activity, as a safe and effective life-style choice, which can modulate gene patterns. Each individual’s genetic background and environmental background are intimately intertwined and interact with lifestyle choices in determining their health status. Increasing evidence shows that environmental and lifestyle factors influence epigenetic mechanisms, such as DNA methylation, histone modifications and microRNA expression and contribute to health or predilection to illness and disease.

Enhancement of environment has also showed results on the influence of exercise on the regulation of the BDNF gene through epigenetic mechanisms, and these results are in harmony with the described influence of other environmental factors on gene expression. For example, Pang and Hannan (2013) described enhancement of cognitive function in animal models of brain disease through combined environmental enrichment and physical activity, Novlovic et al. (2015) showed increased levels of BDNF facilitation of hippocampal synaptic plasticity with environmental enrichment, while Kuzumaki et al. (2011) noted that alterations in environmental stimulation induced epigenetic modification of the BDNF gene. McNair et al. (2007) in using a global proteomics approach, noted changes in the hippocampal proteome following environmental enrichment learning, with protein extracts showing increased expression in functional classes related to energy metabolism, cytoplasmic organization/biogenesis and signal transduction processes.

Several narrative reviews have been published in which the benefits for people engaging in exercise in ‘green’ environments are summarized. In their study comparing the effects on mental and physical wellbeing, of participation in physical activity in natural environments compared with activity indoors, Coon et al. (2011) reported that exercising outdoors was associated with greater feelings of revitalization, positive engagement, increased energy, with decreases in tension, anger, and depression. How genes work together to remodel muscle and brain tissue and promote the many health benefits associated with exercise will take much more research to map out, but understanding that epigenetic changes associate with exercise is an important step in discerning how environmental changes translate to modifications in the cell.

## Summary and future directions

The health benefits of exercise are indisputable in combating age-related risks for disease and disability (Myers et al. 2015; Norton et al. 2014). The take-home message from the published evidence is that exercise training, a lifestyle change that is easily available for most people, can induce changes that affect how we use our genes and improve the quality of our present-day lives, our well-being and our future ageing.

Sullivan and Lachman (2017), in their review concluded that physical activity is broadly beneficial for physical, psychological, and cognitive aspects of health. But, only one in five adults in the US and in most other Western countries, meets the CDC physical activity guidelines of 150 min of aerobic activity and 2 days of muscle strengthening activity per week (Almeida et al. 2014). This trend for inactivity increases with age, as less than half of adults aged 65–74 years and about one-third of adults aged 75 years and older meet current recommendations (Sun et al. 2013). Evidence shows that older participants can benefit as much from exercise programmes as younger groups and have more to gain (Robinson et al. 2017; Barbieri et al. 2015; Hamer et al. 2013; Voss et al. 2013). Making physical activity accessible and feasible for everyone irrespective of age is a public health priority. It is important that physical activity is encouraged but also that there are adequate behavioural and environmental supports. Older and vulnerable groups, who may have physical and cognitive difficulties, which make engagement with exercise difficult, need to be specially supported. Likewise, all age-groups need to be encouraged and facilitated to engage with exercise and physical activities, and make exercise through out life, a life style change.

The association between physical activity and health was recognized, as early as the fifth century BC by the Greek physician Hippocrates, who wrote the following.

All parts of the body, if used in moderation and exercised in labors to which each is accustomed, become thereby healthy and well developed and age slowly; but if they are unused and left idle, they become liable to disease, defective in growth and age quickly.

The motto of the Olympic games since 1894 has been *Citius, Altius, Fortius*, which means—‘Faster, Higher, Stronger’. Most people can never hope or wish to reach an Olympian height of exercise capacity, but most of us can do better and make progress towards being able to be physically active at modest levels—15–30 min a day of brisk walking—which can bring significant health benefits. Inactivity and lack of exercise is shortening lives, and denying people and populations the chance to live better longer lives. The medicine is cheap and effective. It would be wise to follow the insights and example of the GeHA and BELFAST nonagenarian siblings and centenarians and start early and maintain physical activities through out life. Ageing long and well, with combined age-span and health-span, seems increasingly possible.
